# Red Blood Cell Membrane-Camouflaged PLGA Nanoparticles Loaded With Basic Fibroblast Growth Factor for Attenuating Sepsis-Induced Cardiac Injury

**DOI:** 10.3389/fphar.2022.881320

**Published:** 2022-05-17

**Authors:** Xinze Li, Guangliang Hong, Guangju Zhao, Hui Pei, Jie Qu, Changju Chun, Zhiwei Huang, Zhongqiu Lu

**Affiliations:** ^1^ Department of Emergency, The First Affiliated Hospital of Wenzhou Medical University, Wenzhou, China; ^2^ Wenzhou Key Laboratory of Emergency and Disaster Medicine, Wenzhou, China; ^3^ Research Institute of Pharmaceutical Sciences, College of Pharmacy, Chonnam National University, Gwangju, South Korea; ^4^ School of Pharmaceutical Sciences, Wenzhou Medical University, Wenzhou, China

**Keywords:** basic fibroblast growth factor, biomimetic drug carrier, cardiac injury, inflammation, oxidative stress

## Abstract

Cardiac injury is recognized as a major contributor to septic shock and a major component of the multiple organ dysfunction associated with sepsis. Emerging evidence shows that regulation of the intramyocardial oxidative stress and inflammatory response has a promising prospect. Basic fibroblast growth factor (bFGF) exhibits anti-inflammatory and antioxidant properties. In this study, red blood cell membrane-camouflaged poly (lactide-co-glycolide) nanoparticles were synthesized to deliver bFGF (bFGF-RBC/NP) for sepsis-induced cardiac injury. The *in vitro* experiments revealed that bFGF-RBC/NP could protect cardiomyocytes from oxidative and inflammatory damage. In addition, the antioxidant and anti-inflammatory properties of bFGF-RBC/NP against cardiac injury were validated using data from *in vivo* experiments. Collectively, our study used bFGF for the treatment of sepsis-induced cardiac injury and confirmed that bFGF-RBC/NP has therapeutic benefits in the treatment of myocardial dysfunction. This study provides a novel strategy for preventing and treating cardiac injury in sepsis.

## Introduction

Sepsis is a condition caused by a dysregulated host response to an infection, which can lead to severe organ dysfunction (Cecconi et al., 2018), systemic inflammatory response syndrome, and multiple organ dysfunction syndrome (Hollenberg and Singer, 2021). Cardiac injury is a pervasive and fatal complication in sepsis and septic shock (Liu et al., 2020; Xu et al., 2020), occurring in patients with sepsis in intensive care units (Dai et al., 2021). Sepsis-induced cardiac injury may lead to an increased mortality rate of approximately 80%, compared with a 20% mortality rate of sepsis without myocardial dysfunction (Song et al., 2020), despite unceasing advances in research and clinical intervention (Merx and Weber, 2007). Recent studies have demonstrated that sepsis and sepsis-induced cardiac injury have complex pathogenesis (Rudiger and Singer, 2007), including metabolism disorders, inflammatory response, apoptosis, mitochondrial damage, autonomic nervous system dysregulation, and oxidative damage (Li et al., 2019; Zhang et al., 2019; Li et al., 2020). Excessive inflammation and oxidative stress were regarded as the most critical mechanisms. Therefore, effective treatments are needed to improve myocardial function and increase the survival of patients with sepsis-induced cardiac injury and sepsis.

Basic fibroblast growth factor (bFGF), a member of the fibroblast growth factor family that was initially discovered to promote fibroblast growth (Belov and Mohammadi, 2013; Babina and Turner, 2017), is one of the multiple factors that can stimulate and support proliferation, migration, and differentiation (Yayon and Klagsbrun, 1990). To date, promising evidence has revealed that bFGF can promote heart repair *via* pro-angiogenetic, anti-apoptotic, and pro-proliferative mechanisms with clinical safety (Virag et al., 2007). For example, previous studies reported a ROS-sensitive hydrogel was synthesized to deliver bFGF for myocardial repair (Li et al., 2021b), whereas Fan et al. (2019) developed a bFGF delivery system to attenuate cardiac fibrosis. In addition, a study expounded the benefit of bFGF in sepsis-induced acute lung injury by the suppressive effect on inflammation *via* the NF-κB pathway (Pan et al., 2020). Therefore, the application of bFGF in sepsis-induced cardiac injury is promising and meaningful. However, several barriers, such as rapid diffusion, poor biostability, and short half-life, limited the clinical efficacy of bFGF (Deveza et al., 2012). Hence, a safe and effective delivery strategy to achieve sustained-release and bioactivity retention of bFGF is essential and urgent.

Various delivery systems, such as hydrogel (Mai et al., 2020), nanoparticles (Liu et al., 2013), liposomes (Xu et al., 2017), and microsphere (Choi et al., 2019), have emerged to solve these problems. Nanoparticles have attracted much attention as a potential vehicle for the delivery of bFGF genes and proteins (Zhao et al., 2014). As a copolymer of polylactide (PLA) and polyglycolide (PGA), poly (lactide-co-glycolide) (PLGA) has been widely used in nanotechnology since 1970 (Chereddy et al., 2018). PLGA, known for its biocompatibility, biodegradability, and low toxicity, has been approved by the FDA for human use (Crotts and Park, 1997). When decorated by a PLGA microsphere, sustained release of bFGF can promote the proliferation and differentiation of various cells (Kim et al., 2020). In addition, therapeutic functions of PLGA microspheres in the delivery of a controlled-release matrix of bFGF and regeneration of the sciatic nerve have been confirmed (Si et al., 2017). Recently, novel strategies have been developed for longer circulation and less immune recognition of nanoparticles; they include modification with polyethylene glycol (PEG), cell-penetrating peptides, and cell-derived membranes of macrophages, dendritic cells, stems cells, and red blood cells (RBCs) (Shreffler et al., 2019; Wu et al., 2021). Particularly, cell membranes of RBCs have been of much focus due to the excellent biocompatibility, accessibility, and long half-life of RBCs, which are the most abundant cells in the blood (Gao et al., 2017). The advances in cellular and molecular biology have revealed that RBCs protect nanoparticle-based drugs from blood clearance and deliver them into strategic organs (Guo et al., 2015). This property is due to the self-recognition ability of the homologous RBCs, which are arbitrated by various proteins, such as CD47, residing on cell membranes (Oldenborg et al., 2000). For instance, Cheng et al. revealed that the RBC membrane may inspire nanoparticle therapy in acute liver failure (Liang et al., 2018). Sun et al. constructed a long circulatory and sustained release system using RBC membrane-coated nanoparticles to protect ischemic myocardium (Huang K. et al., 2021). Notably, although numerous biomimetic nanoparticles have been explored for the delivery of bFGF, their application in sepsis-induced cardiac injury has been rarely reported.

In this study, bFGF was encapsulated into PLGA nanoparticles and wrapped with RBC membranes (bFGF-RBC/NP) to overcome the aforementioned obstacles and optimize therapeutic effects. Thereafter, bFGF-RBC/NP was characterized, and its therapeutic efficacy in sepsis-induced cardiac injury was evaluated *in vitro* and *in vivo*. We hypothesized that the controlled release and bioactive retention of bFGF-RBC/NP played a pivotal role, and coating with the RBC membrane could enable them to evade the phagocytic system and achieve a long half-life in circulation. For further investigation, we examined the relevant indicators and found that bFGF-RBC/NP exerted good therapeutic effects on sepsis-induced cardiac injury through its antioxidative and anti-inflammatory capacities.

## Materials and Methods

### Materials, Cells, and Animals

bFGF was provided by the Key Laboratory of Biotechnology Pharmaceutical Engineering of Wenzhou Medical University (Wenzhou, China). PLGA, Poloxamer 407 (P407), and polyvinyl alcohol (PVA) were provided by Sigma (Shanghai, China). Cell Counting Kit 8 (CCK-8) was obtained from Yeasen Biotechnology Co., Ltd. (Shanghai, China). The superoxide dismutase (SOD) activity kit, the glutathione (GSH) assay kit, glutathione peroxidase (GPX) assay kit, high mobility group protein 1 (HMGB1) enzyme-linked immunosorbent assay (ELISA) kit, and lipopolysaccharides (LPS) were purchased from Solarbio Science & Technology Co., Ltd. (Beijing, China). DMEM, fetal bovine serum (FBS), bovine serum albumin (BSA), and trypsin were obtained from Gibco (CA, USA). The bicinchoninic acid (BCA) protein assay kit, nitric oxide (NO) assay kit, lactate dehydrogenase (LDH) assay kit, malondialdehyde (MDA) assay kit, catalase (CAT) assay kit, caspase-3 activity assay kit, TUNEL apoptosis assay kit, Annexin V-FITC apoptosis detection kit, and reactive oxygen species (ROS) assay kit were bought from Beyotime Biotechnology (Shanghai, China). Interleukin-1β (IL-1β, 70-EK201B/3-96, and 70-EK301B/3-96), interleukin-6 (IL-6, 70-EK206/3-96, and 70-EK306/3-96), tumor necrosis factor-α (TNF-α, 70-EK282/4-96, and 70-EK382/3-96), and bFGF (70-EK1F03-96) enzyme-linked immunosorbent assay (ELISA) kits were supplied by Multisciences Biotech Co., Ltd. (Hangzhou, China). The HMGB1 antibody (ab79823), CD47 antibody (ab214453), iNOS antibody (ab283655), and CD68 antibody (ab125212) were provided by Abcam (MA, USA). The creatine kinase isoenzyme MB (CK-MB) and myeloperoxidase (MPO) assay kits were supplied by Jiancheng Bioengineering Institute (Nanjing, China). Cell culture plates, coverslips, and centrifuge tubes were obtained from NEST Biotechnology Co., Ltd. (Wuxi, China). All other chemicals and buffer solutions were of analytical grade.

The rat embryonic cardiomyocytes (H9c2 cells) were obtained from the Shanghai Institute of Biochemistry and Cell Biology (Shanghai, China). The cells were cultured in DMEM supplemented with 10% (v/v) FBS and 1% (v/v) penicillin/streptomycin in a cell incubator.

Male C57BL/6 mice (6–8 weeks old, 22–26 g) were purchased from the Shanghai Laboratory Animal Center (Shanghai, China). The mice were acclimated to a pathogen-free animal facility for 7 days and then randomly grouped for experiments. All procedures involving animals were performed in accordance with the National Institutes of Health Guidelines for the Care and Use of Laboratory Animals.

### Preparation of the Red Blood Cell Membrane

The red blood cell (RBC) membrane was prepared, as reported (Rao et al., 2015). Whole blood was collected from C57BL/6 mice with EDTA spray-coated tubes and then centrifuged at 1,000 rpm for 5 min to remove the plasma and buffy coat. The harvested RBCs were washed with cold PBS three times to remove the residual serum. Subsequently, RBCs were resuspended into diluted PBS in an ice bath for 30 min to induce membrane rupture. Subsequently, the solution was centrifuged at 15,000 rpm for 5 min, and the light pink pellet was collected. After washing with PBS, the collected samples were resuspended in water and sonicated for 15 min using a bath sonicator (100 W), followed by a sequential extrusion through 400 and 200 nm polycarbonate porous membranes with a mini extruder. The harvested RBC membranes were stored in water at 4°C.

### Preparation of bFGF-NP

bFGF-NP was synthesized using a modified double emulsion solvent evaporation technique, as in a previous study (Zhao et al., 2021). Initially, 0.1 g PLGA and 0.01 g P407 were dissolved in 2 ml dichloromethane to form an oil phase. Thereafter, the bFGF solution (0.5 mg/ml) was added to the oil phase and sonicated using a probe ultrasonic instrument (50 W, 5 min) to obtain the primary emulsion. Subsequently, the emulsion was added to 1% (v/v) PVA solution in PBS, thus forming double emulsion. After stirring for 12 h, the organic solvent was removed using a rotary evaporator. The remaining suspension was filtered through a porous glass filter, freeze-dried using a freeze dryer, and stored at 4°C.

### Preparation of bFGF-RBC/NP

bFGF-RBC/NP was synthesized using an extrusion method, as described in a previous study (Wang et al., 2019). The RBC membrane (50 μl) and bFGF-NP (4 mg) were mixed and ultrasonicated for 5 min (100 W). Thereafter, the mixture was extruded 10 times through a 200-nm porous polycarbonate membrane using an Avanti Mini-Extruder to obtain bFGF-RBC/NP.

### Characterization of bFGF-RBC/NP

The membrane proteins were characterized by sodium dodecyl sulfate–polyacrylamide gel electrophoresis (SDS-PAGE). Western blot was used to analyze CD47 in the RBC lysate, RBCs, and bFGF-RBC/NP. The results were observed using a ChemiDoc-XRS imaging system.

The size, polydispersity index (PDI), and zeta potential of bFGF-NP and bFGF-RBC/NP were measured using a dynamic light scattering detector (Litesizer, Anton Paar, Austria). Thereafter, bFGF-RBC/NP was incubated in PBS and subsequently PBS with 10, 20, and 40% FBS. The diameter and PDI changes were monitored for 7 days to analyze the stability under physiological conditions. Subsequently, the morphology was observed under a transmission electron microscope (TEM, JEM-100CX, Japan). The encapsulation efficiency of bFGF-RBC/NP containing various concentrations of bFGF was determined.

*In vitro* release of bFGF was evaluated using the dynamic dialysis method. bFGF, bFGF-NP, and bFGF-RBC/NP were placed into dialysis tubes (100 kDa) and immersed in PBS at 37°C for 72 h under horizontal shaking at 100 rpm. PBS was collected and replaced with an equal volume of fresh PBS periodically. bFGF in PBS was detected using the ELISA kit.

### Cytocompatibility of bFGF-RBC/NP

Cell viability was assessed using the CCK-8 kit. H9c2 cardiomyocytes were seeded into 96-well plates and incubated in a humidified 5% CO_2_ incubator at 37°C for 24 h. Thereafter, H9c2 cells were treated with different concentrations of bFGF, bFGF-NP, and bFGF-RBC/NP (1–200 ng) for another 24 h. Subsequently, 10 μl of the 2-[2-methoxy-4-nitrophenyl]-3-[4-nitrophenyl]-5-[2,4-disulfophenyl]-2H-tetrazolium (WST-8) reagent was added to each well and co-cultured for 2 h. Finally, the absorbance of the 96-well plates was read at 450 nm using a microplate reader (Tecan Safire, Germany).

### Cellular Uptake of bFGF-RBC/NP

To observe the cellular uptake of bFGF-RBC/NP, Cy5 was conjugated to bFGF, according to the method described in a previous study (Hwang et al., 2010). H9c2 cells were seeded into 12-well plates, incubated with Cy5-labeled bFGF and bFGF-RBC/NP for 4 h, and washed with PBS three times. The cells were stained with 4’,6-diamidino-2-phenylindole (DAPI) and observed under a laser scanning confocal microscope (CLSM, Nikon, Japan). In addition, the cells were collected, and intracellular fluorescence was analyzed using a flow cytometer.

### *In Vitro* Anti-Apoptotic, Antioxidant, and Anti-Inflammatory Capacities of bFGF-RBC/NP

To evaluate the anti-apoptotic property of bFGF-RBC/NP, flow cytometry and caspase-3 activity assay were performed. H9c2 cells were pretreated with LPS (1 μg/ml) for 24 h and then incubated with bFGF and bFGF-RBC/NP (50 ng/ml) for another 24 h. Subsequently, the cells were detached with 0.25% trypsin-EDTA, washed with PBS, stained with Annexin V-FITC and PI, and collected for flow cytometry to assess cell apoptosis. In addition, the caspase-3 activity in H9c2 cells was detected using a caspase-3 assay kit.

H9c2 cells were pretreated with LPS (1 μg/ml) and incubated with bFGF and bFGF-RBC/NP (50 ng/ml) for the subsequent experiments. Intracellular ROS production was determined using a ROS fluorescent probe and observed under the fluorescence microscope (Olympus Corp., Tokyo, Japan). The cell viability was evaluated using the CCK-8 kit. In addition, the CAT, SOD, MDA, and NO levels in cells of each group were detected using the appropriate reagent kits. To examine the anti-inflammatory effects of bFGF-RBC/NP, the cell supernatants were collected, and the levels of inflammatory factors (IL-6 and TNF-α) were measured using ELISA kits.

### Sepsis-Induced Cardiac Injury Model

The septic cardiomyopathy model was established using a cecal ligation and puncture (CLP) procedure, as described in the literature (Rittirsch et al., 2009). Mice were anesthetized, and a longitudinal skin midline incision was made with a scalpel. The cecum was ligated and punctured twice with a 21-gauge needle. After feces were extruded, the cecum was returned to the abdominal cavity, and the wound was closed by carefully running sutures on the abdomen.

The mice were randomly divided into four groups (*n* = 3): 1) Control+saline (sham group), 2) CLP+saline, 3) CLP+bFGF (100 μg/kg), and 4) CLP+bFGF-RBC/NP (100 μg/kg). All animals were treated with different interventions through the tail vein once daily for 7 days before CLP. After fasting for 24 h, mice received intravenous administration of bFGF or bFGF-RBC/NP. After 2 h, mice underwent CLP operation. After 24 h, the blood and major organs were collected for subsequent analysis.

### Echocardiography

The cardiac function was assessed by echocardiography 24 h after CLP operation. The images were captured with a visual sonics (Vevo 3100) high-resolution imaging system. The parameters for evaluating the cardiac function, left ventricular end-diastolic diameter (LVEDD), left ventricular end-systolic diameter (LVESD), left ventricular ejection fraction (EF), and left ventricular fractional shortening (FS) of each group were measured.

### Biodistribution and Pharmacokinetics of bFGF-RBC/NP

To compare the *in vivo* distribution of bFGF and bFGF-RBC/NP, the CLP mice were injected with Cy5-labeled bFGF (100 μg/kg) or Cy5-labeled bFGF-RBC/NP (100 μg/kg). After 90 min, the heart tissues of each group were collected and observed by using the imaging system (PerkinElmer, MA, USA).

In addition, the CLP mice were divided into two groups randomly and administered with bFGF (100 μg/kg), bFGF-NP (100 μg/kg), or bFGF-RBC/NP (100 μg/kg). At the presetting time intervals (1, 5, 10, and 30 min and 1, 2, 4, 8, 12, and 24 h) after injection, the blood samples were collected, and the levels of bFGF in blood were measured by using the ELISA kit.

### Histological Analysis

The tissues were collected, cut into appropriate blocks, and fixed in 4% paraformaldehyde solution for 24 h. Samples were dehydrated, embedded, and sectioned into slices, following the established procedure (Huang Z. et al., 2021). For immunohistochemistry (IHC) and immunofluorescence (IF) analysis, the sliced samples were deparaffinized, incubated with 3% H_2_O_2_ for 10 min to block endogenous peroxidase, and heated in citrate antigen retrieval solution for antigen retrieval. After blocking with 5% BSA, the sections were incubated with CD68 (1:200), HMGB1(1:400), and iNOS (1:50) antibodies overnight at 4°C. After washing with PBS three times, the sections were incubated with the horseradish peroxidase polymer secondary antibody for 1 h at 37°C. For IF analysis, the sections were directly stained with DAPI and observed under a confocal laser scanning microscope. IHC and TUNEL staining were performed, according to the manufacturer’s instructions. All stained tissue sections were observed under an optical microscope (Olympus Corp., Tokyo, Japan).

### *In Vivo* Therapeutic Effects of bFGF-RBC/NP

The heart in each group was weighed, and the heart weight-body weight ratio was calculated. The LDH and creatine kinase isoenzyme (CK-MB) levels in the serum were estimated. The levels of GSH, GPX, CAT, SOD, MPO, and MDA in heart tissues were determined using the appropriate kits. ELISA kits were used to detect the concentrations of IL-1, IL-6, TNF-α, and HMGB1 in heart tissues.

### Statistical Analyses

Student’s t-test and one-way analysis of variance assay were performed for statistical analysis. Data are expressed as the mean ± SD in independent experiments. *p < 0.05 and **p < 0.01 indicated statistical significance.

## Results

### Characterization of bFGF-RBC/NP

**FIGURE 1:**
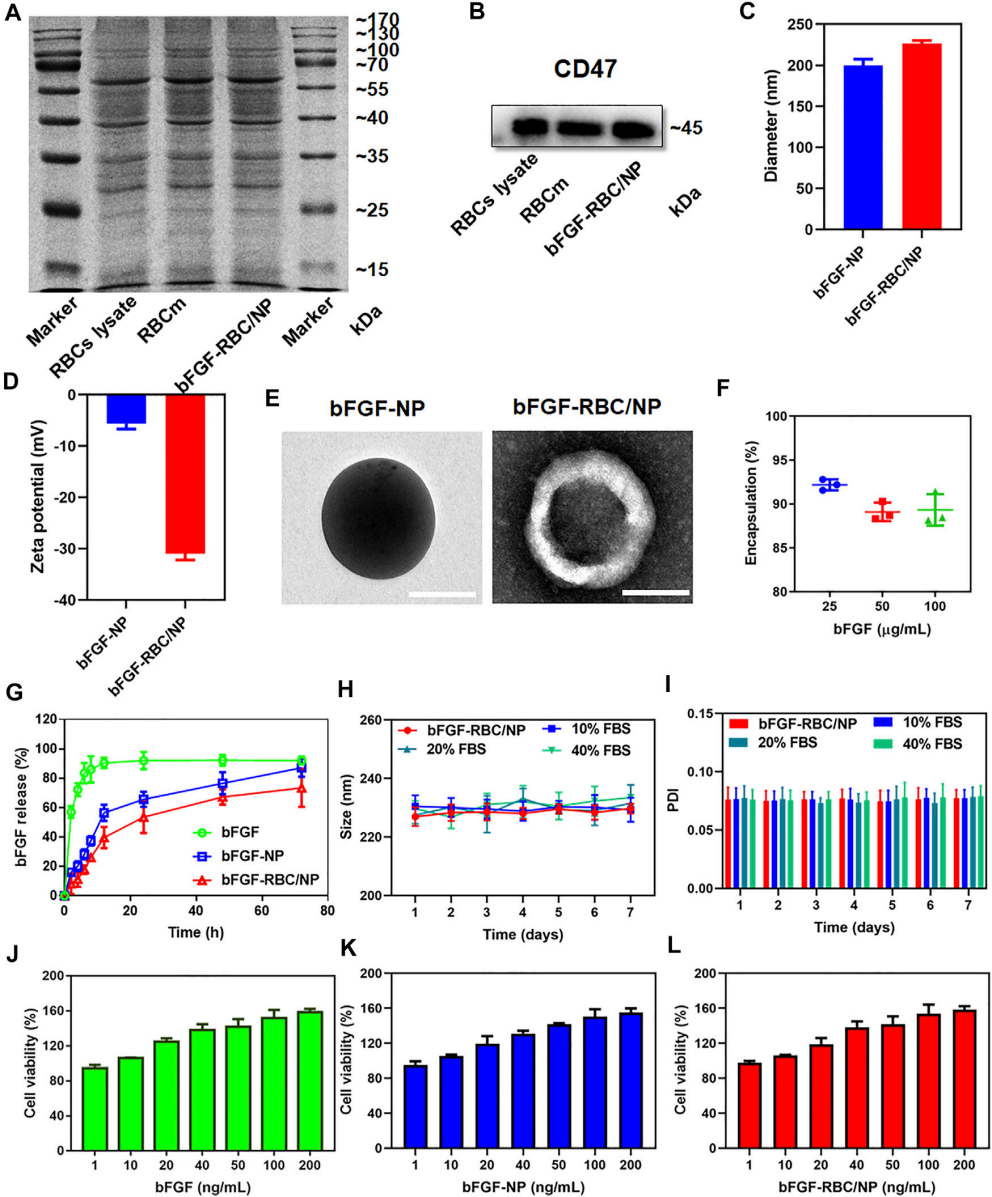
Characterization of bFGF-RBC/NP. **(A)** SDS-PAGE of red blood cell (RBC) lysate, RBC membrane, and bFGF-RBC/NP. **(B)** Western blot analysis of CD47 in the RBC lysate, RBC membrane, and bFGF-RBC/NP. **(C)** Diameter, **(D)** zeta potential, and **(E)** TEM of bFGF-NP and bFGF-RBC/NP. **(F)** Encapsulation efficiency and **(G)** release of bFGF-RBC/NP. **(H)** Size and **(I)** PDI of bFGF-RBC/NP in PBS containing 0, 10, 20, and 40% FBS for 7 days. Cell viability after cell incubation with different concentrations of **(J)** bFGF, **(K)** bFGF-NP, and **(L)** bFGF-RBC/NP. Data are expressed as the mean ± SD (*n* = 3). Scale bar = 100 nm.

To overcome bFGF’s limitations of instability and rapid degradation right after being administered, bFGF-RBC/NP was synthesized following three steps: I) extraction of RBC membrane-derived vehicles, II) preparation of bFGF-NP, and III) cloaking of the RBC membrane onto bFGF-NP. Subsequently, a series of experiments were performed to characterize bFGF-RBC/NP. As shown in Figure 1A, SDS-PAGE analysis toward a series of membrane protein markers indicated that bFGF-RBC/NP was coincident in protein bands with the RBC lysate and RBC membrane, suggesting good retention of characteristic proteins inherited from the RBC membrane. Western blot results demonstrated the presence of CD47 on the surface of bFGF-RBC/NP (Figure 1B).

Subsequently, we evaluated the particle size distribution and zeta potential of bFGF-NP and bFGF-RBC/NP, as exhibited in Figures 1C,D. The hydrodynamic diameter of bFGF-RBC/NP increased from 199.7 ± 7.77 nm (bFGF-NP) to 226.47 ± 3.65 nm after coating with the RBC membrane. Accordingly, the zeta potential of bFGF-RBC/NP changed from –5.67 ± 1.03 mV (bFGF-NP) to –31 ± 1.21 mV, due to the negative charge of the RBC membrane. In addition, we analyzed the morphology of bFGF-NP and bFGF-RBC/NP under a transmission electron microscope (TEM). Both bFGF-NP and bFGF-RBC/NP had a uniform spherical morphology with a smooth and non-porous surface (Figure 1E). Particularly, bFGF-RBC/NP showed a clear core-shell nanostructure with a bFGF-NP (core) and RBC membrane (shell), indicating successful coating of the RBC membrane upon bFGF-RBC/NP.

Additionally, the encapsulation efficiency and drug release of bFGF-RBC/NP were measured using ELISA kits. As described in Figure 1F, the bFGF loading efficiency varied from 92.18 ± 0.62% to 89.33 ± 1.80% as the concentration of bFGF increased from 25 to 100 μg. *In vitro* release profiles were recorded over a course of 72 h. The results showed that 92.73 ± 0.63% of bFGF had been released from the bFGF solution at the end time; approximately 90% of bFGF was detected within 8 h. By contrast, the obvious sustained release of bFGF from bFGF-NP and bFGF-RBC/NP was observed, and no instant release condition was present within the first 2 h. In the presence of an RBC membrane, bFGF-RBC/NP showed a slower release of bFGF than bFGF-NP within 24 h (Figure 1G).

The stability of bFGF-RBC/NP was investigated under different circumstances (Figures 1H,I). To simulate the physiological condition in circulation, bFGF-RBC/NP was incubated in PBS and PBS with 10, 20, and 40% FBS. Thereafter, the sizes and PDI were examined for 7 days. No significant changes were recorded, indicating that bFGF-RBC/NP could preserve the stable nanostructure, while exposed to the serum. However, the size and PDI of bFGF-NP underwent significant fluctuations and gradually increased with observation time (Supplementary Figures S1A,B), which implied that its instability and the coating of the RBC membrane facilitated the nanoparticles to remain stable.

The viability of H9c2 cells treated with different concentrations of bFGF, bFGF-NP, and bFGF-RBC/NP were measured (Figures 1J–L). The viability of cells increased as the concentration changed from 1 to 200 ng/ml, demonstrating that bFGF-RBC/NP was less toxic to H9c2 cells and promoted cell proliferation, owing to the efficacy of bFGF.

### *In Vitro* Cellular Uptake and Anti-Apoptotic Capacity of bFGF-RBC/NP

**FIGURE 2:**
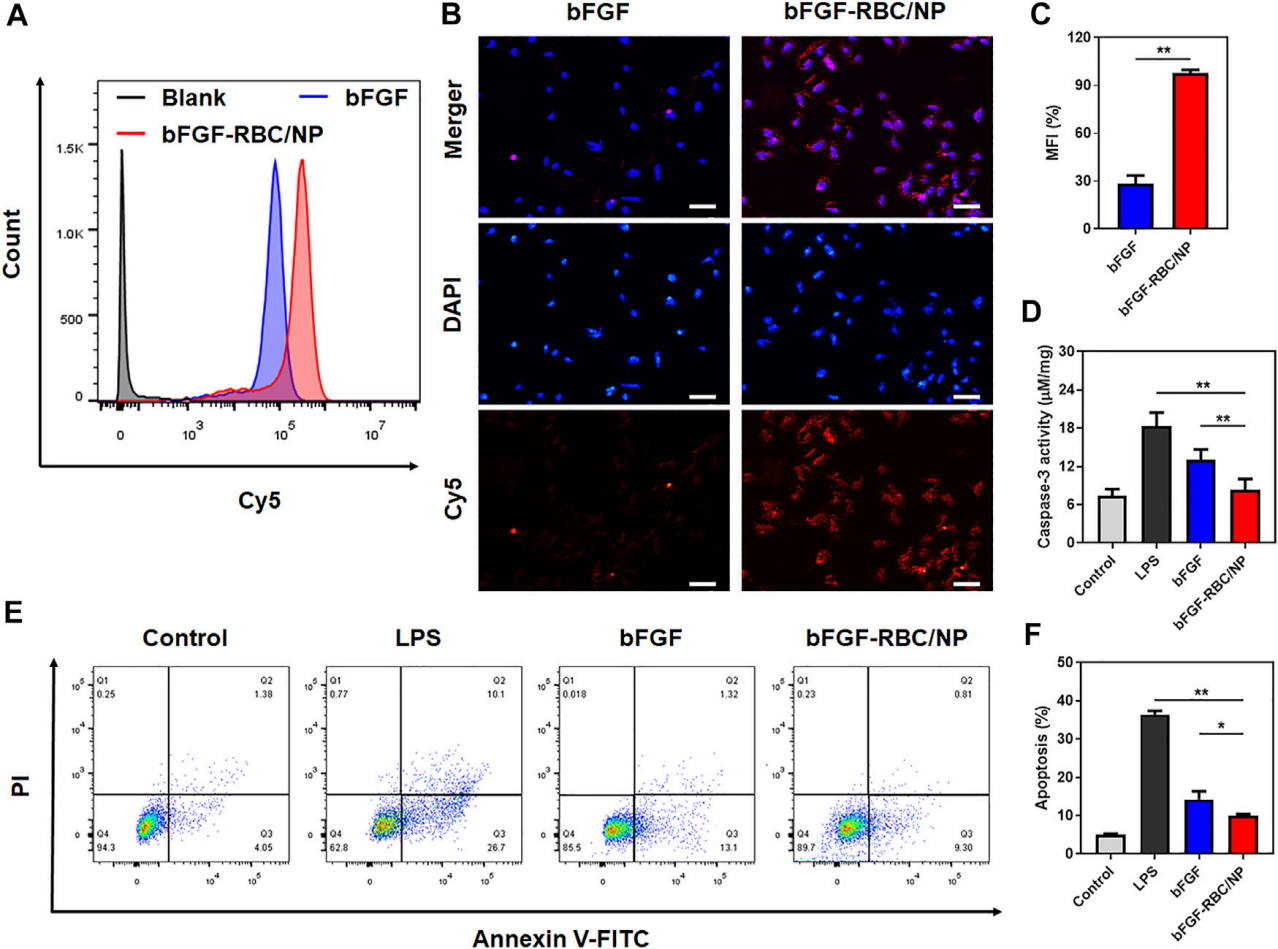
*In vitro* cellular uptake and anti-apoptotic capacity of bFGF-RBC/NP. The cells were treated with bFGF and bFGF-RBC/NP (50 ng/ml) for 4 h, and the cellular uptake was analyzed by **(A)** flow cytometry and **(B)** laser scanning confocal microscopy. **(C)** Mean fluorescent intensity (MFI) of each group. The LPS-stimulated (1 μg/ml) H9c2 cells were treated with bFGF and bFGF-RBC/NP (50 ng/ml) for 24 h. **(D)** Caspase-3 activity of each group. **(E)** Apoptosis was assessed by flow cytometry and **(F)** quantitative analysis. Data are expressed as the mean ± SD (*n* = 3). Scale bar = 50 um. *p < 0.05 and **p < 0.01.

To study the cellular uptake of bFGF-RBC/NP, bFGF and bFGF-RBC/NP were labeled with Cy5 in advance and incubated with H9c2 cells for 4 h. As shown in Figures 2A,B, the bFGF-RBC/NP group exhibited much stronger fluorescent intensities, consistent with a noticeable red fluorescence distributed inside the cytoplasm than the bFGF group (Figure 2C). Subsequently, we investigated the anti-apoptotic capacity of bFGF-RBC/NP. H9c2 cells were pre-stimulated with LPS (1 μg/ml) and incubated with bFGF and bFGF-RBC/NP (50 ng/ml) for 24 h. The caspase-3 activity was measured, and LPS induced a significant increase in the caspase-3 activity. The bFGF-RBC/NP group showed reduced caspase-3 activity (Figure 2D). Additionally, the cells were detected by flow cytometry after staining with Annexin V-FITC and PI. As illustrated in Figures 2E,F, the apoptotic cells were approximately 40% in the LPS group, whereas after treatment with bFGF and bFGF-RBC/NP, the apoptosis rate decreased to <15%. These results indicate that bFGF-RBC/NP had a better anti-apoptotic capacity than bFGF with an apoptosis rate of approximately 10%, indicating that biomimetic nanoparticles enhanced the protective capacity against LPS-induced damage of bFGF.

### *In Vitro* Antioxidant and Anti-Inflammatory Effects of bFGF-RBC/NP

**FIGURE 3:**
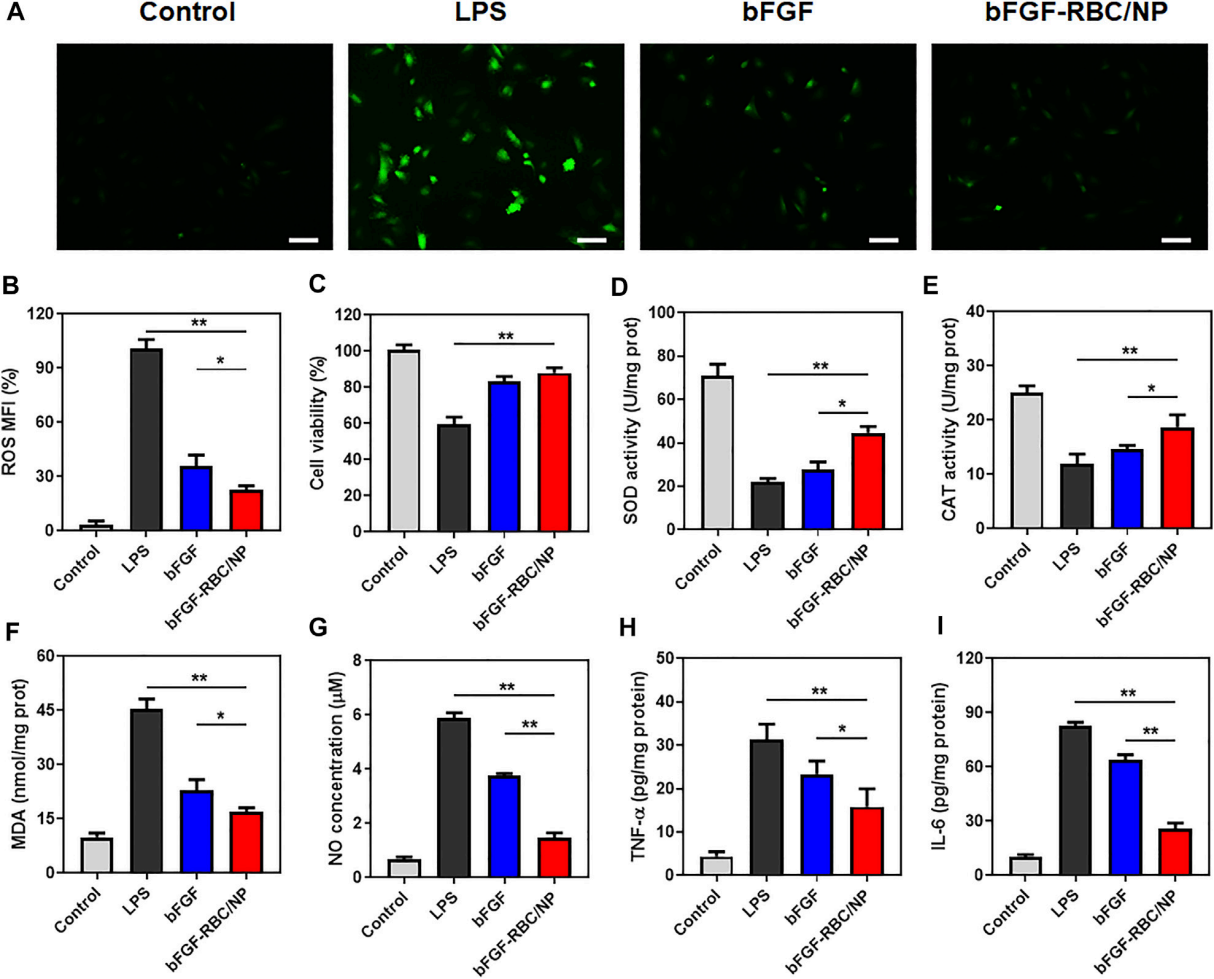
*In vitro* antioxidative and anti-inflammatory efficacy of bFGF-RBC/NP. LPS-stimulated (1 μg/ml) H9c2 cells were treated with bFGF and bFGF-RBC/NP (50 ng/ml) for 24 h. **(A)** Intracellular reactive oxygen species (ROS) of each group. **(B)** Semi-quantitative analysis of the mean fluorescence intensity (MFI) of ROS. **(C)** Cell viability of each group. The levels of **(D)** SOD, **(E)** CAT, **(F)** MDA, and **(G)** NO in cells of each group. The contents of **(H)** TNF-α and **(I)** IL-6 in the supernatant. Data are expressed as the mean ± SD (*n* = 3). Scale bar = 50 um. *p < 0.05 and **p < 0.01.

Oxidant stress and inflammatory response play an important role in the development of cardiac injury during sepsis. To determine the anti-oxidative and inflammatory efficacy of bFGF-RBC/NP, H9c2 cells were pre-stimulated with LPS (1 μg/ml) and incubated with bFGF and bFGF-RBC/NP (50 ng/ml) for the subsequent experiments. We evaluated the ROS production using a fluorescent probe. The LPS group exhibited a far stronger fluorescent intensity than others. Contrarily, bFGF-RBC/NP decreased ROS generation after LPS injury and exhibited stronger resistance against ROS than bFGF (Figures 3A,B). The cell viability was assessed using CCK-8. Evidently, H9c2 cell viability declined after exposure to LPS, but it was enhanced after treatment with bFGF-RBC/NP (Figure 3C). Accordingly, bFGF-RBC/NP distinguishingly boosted SOD and CAT activities (Figures 3D,E) and lowered the productions of MDA and NO (Figures 3F,G). In general, bFGF-RBC/NP significantly improved intracellular antioxidant enzyme activities and reduced the harmful substances attributed to oxidant stress. In addition, the supernatant of each group was collected to measure the level of pro-inflammatory cytokines, TNF-α and IL-6. As shown in Figures 3H,I, bFGF and bFGF-RBC/NP downregulated the expression of inflammatory cytokines, whereas a sharp rise occurred in the LPS group. These results suggested that bFGF-RBC/NP could adequately protect H9c2 cells from the damage caused by oxidative stress and inflammatory response.

### The Assessments of Cardiac Function and Biodistribution of bFGF-RBC/NP

**FIGURE 4:**
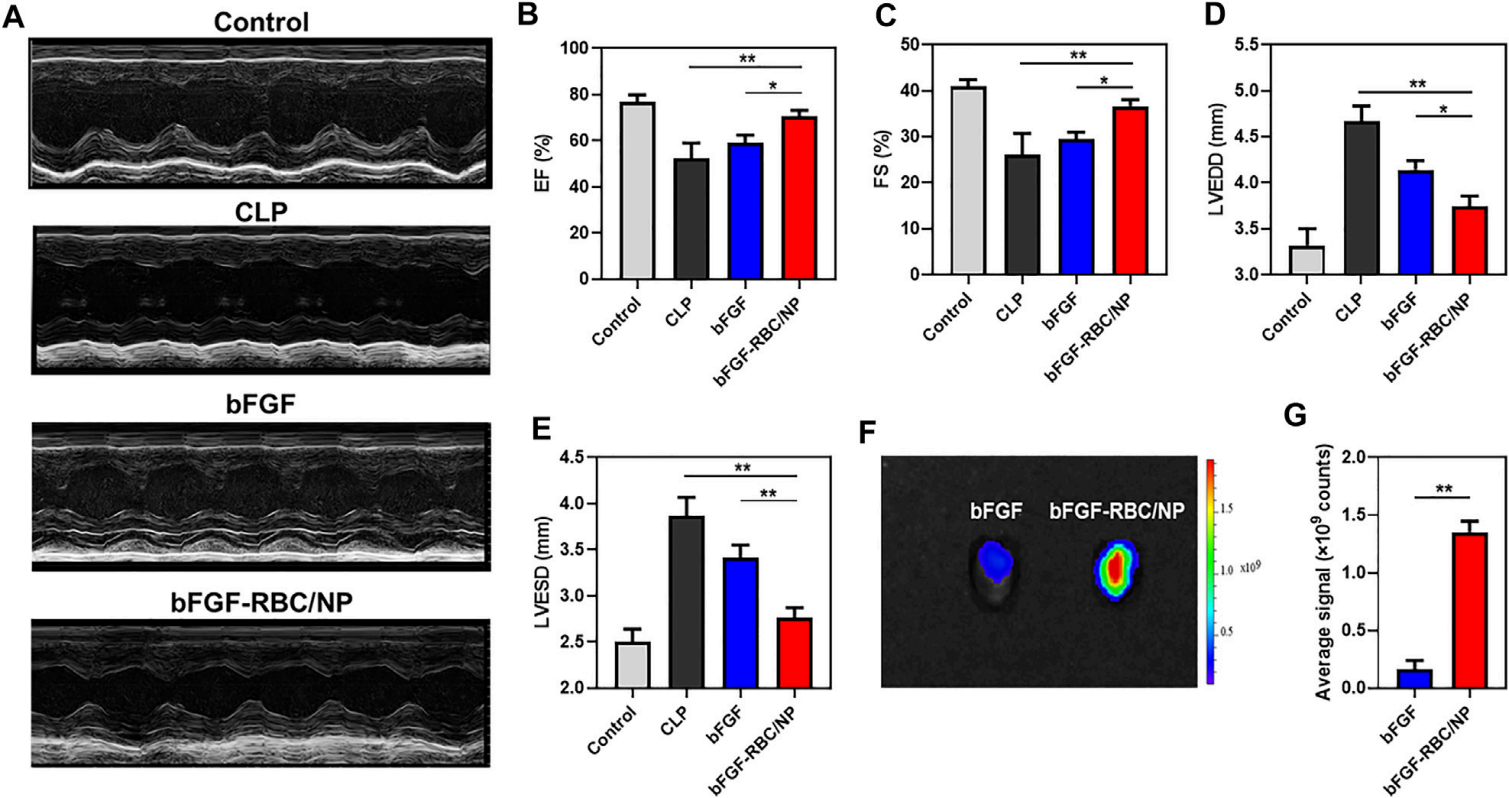
Assessment of cardiac function and distribution of bFGF-RBC/NP in the heart. **(A)** Representative echocardiographic images of each group. The levels of **(B)** left ventricular ejection fraction (EF), **(C)** left ventricular fractional shortening (FS), **(D)** left ventricular end-diastolic diameter (LVEDD), and **(E)** left ventricular end-systolic diameter (LVESD). **(F)** Fluorescent distribution of Cy5-labeled bFGF and Cy5-labeled bFGF-RBC/NP in the heart of mice 90 min after bFGF or bFGF-RBC/NP injection. **(G)** Quantitative analysis of the fluorescent intensity in the heart. Data are expressed as the mean ± SD (*n* = 3). *p < 0.05 and **p < 0.01.

To investigate the effects of bFGF-RBC/NP on cardiac function in CLP mice, echocardiography was employed to analyze the parameters of cardiac function (Figure 4A). The values of EF and FS were decreased in the CLP group compared to that in the control group. In contrast, bFGF-RBC/NP increased the EF and FS levels in CLP mice (Figures 4B,C). Additionally, LVEDD and LVESD were significantly increased in the LPS group compared to the control group, whereas bFGF-RBC/NP significantly reduced the increases in LVEDD and LVESD of CLP mice (Figures 4D,E). These results showed that bFGF-RBC/NP effectively improved the cardiac function in mice with sepsis. Furthermore, the cardiac delivery of bFGF and bFGF-RBC/NP *in vivo* was also evaluated. As shown in Figure 4F, the fluorescent intensity in the heart of bFGF-RBC/NP was stronger than that of the bFGF group. The results of the quantitative analysis showed that the fluorescent signal of the bFGF-RBC/NP group was much higher than that of the bFGF group (Figure 4G), which meant that higher concentrations of bFGF in the heart of the bFGF-RBC/NP group due to camouflage of RBC. The pharmacokinetic results showed that after injection, the concentration−time curves of bFGF and bFGF-NP showed the levels of bFGF and bFGF-NP declined rapidly within 4 h, while the decline of concentration of bFGF-RBC/NP *in vivo* was relatively slow (Supplementary Figure S2), which implied that bFGF-RBC/NP could be retained in the systemic circulation for a long time.

### Therapeutic Effects of bFGF-RBC/NP in Sepsis-Induced Cardiac Injury

**FIGURE 5:**
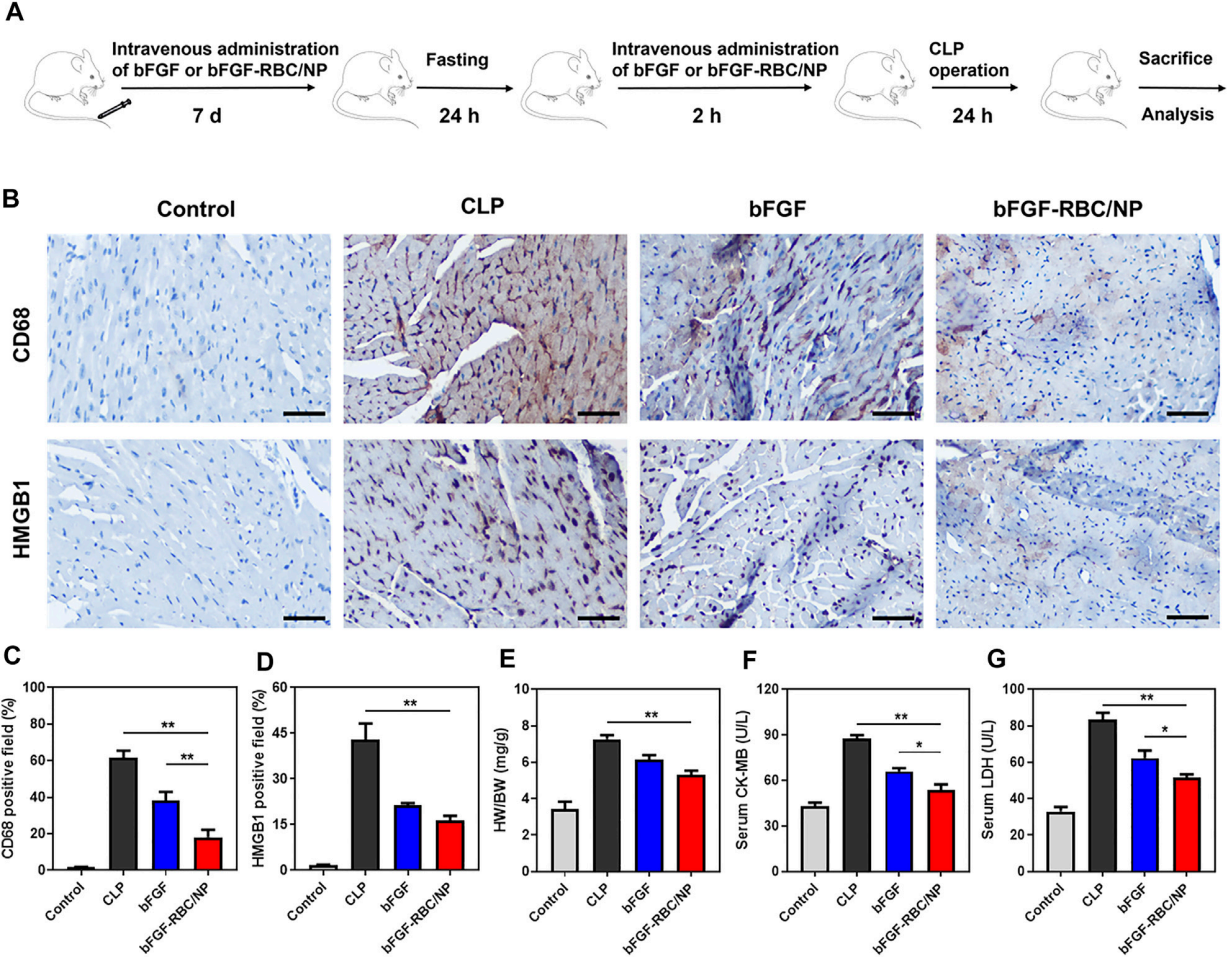
Therapeutic effects of bFGF-RBC/NP. **(A)** Timeline of the experimental procedure. **(B)** Representative immunohistochemical staining results of CD68 and HMGB1 in heart tissues. The quantitation of positive cells of **(C)** CD68 and **(D)** HMGB1 in each group. **(E)** Heart weight/bodyweight (HW/BW) index of each group. **(F)** CK-MB and **(G)** LDH levels in serum. Data are expressed as the mean ± SD (*n* = 3). Scale bar = 50 um. *p < 0.05 and **p < 0.01.

We established a sepsis-induced cardiac injury model by CLP surgery to identify the protective effects of bFGF-RBC/NP *in vivo*, according to the experimental procedure illustrated in Figure 5A. The mice were sacrificed 24 h after the CLP procedure, and the heart and blood samples were collected. Our previous pre-experimental results showed that bFGF-NP exhibited almost identical effects compared to free bFGF (Supplementary Figure S2), which indicated that nanoparticulation of bFGF with PLGA alone cannot yield satisfactory results. Thus, in a follow-up study, we compared the *in vivo* therapeutic effects of bFGF and bFGF-RBC/NP. The IHC staining results demonstrated that bFGF-RBC/NP could alleviate cardiac injury by antagonizing the inflammatory cytokines. We mainly focused on HMGB1, which directly impaired the myocardial function in sepsis, and the expression of HMGB1 was remarkably increased in the CLP model. As one of the major sources of HMGB1, the macrophages were involved in vast inflammatory reactions. Therefore, the macrophage marker CD68 antibody was used to analyze macrophage aggregation in the heart. CD68-positive macrophages gradually declined when treated with bFGF and bFGF-RBC/NP (Figure 5B), and quantitative analyses of HMGB1 and CD68 expressions were conducted (Figures 5C,D). In addition, the heart weight index (heart weight/bodyweight, HW/BW) of each group was calculated. The value of HW/BW in the CLP group was abnormally high, whereas that of the bFGF and bFGF-RBC/NP groups was lower (Figure 5E). Subsequently, we measured LDH and CK-MB levels in sera for each group; LDH and CK-MB were regarded as myocardial injury marker enzymes (Figures 5F,G). The drastically increased levels of LDH and CK-MB in sepsis were reversed after administration with bFGF and bFGF-RBC/NP. Importantly, these data revealed that bFGF-RBC/NP exerted better therapeutic effects than bFGF.

**FIGURE 6:**
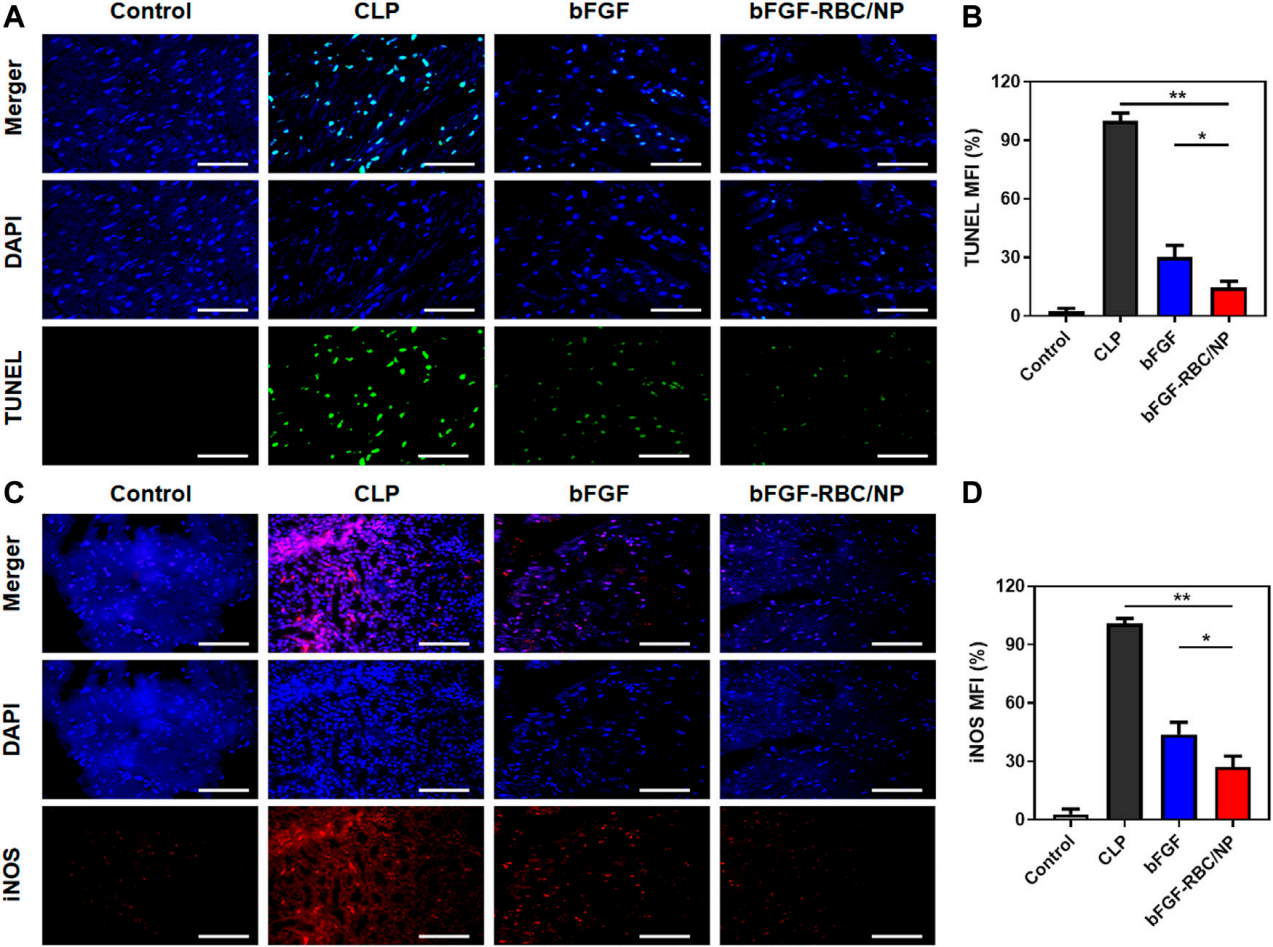
Fluorescent staining analysis of bFGF-RBC/NP. **(A)** TUNEL and **(C)** immunofluorescent staining of iNOS in heart tissues. The semi-quantitative analysis of the mean fluorescent intensity of **(B)** TUNEL and **(D)** iNOS of each group. Data are expressed as the mean ± SD (*n* = 3). Scale bar = 50 um. *p < 0.05 and **p < 0.01.

To assess the protective effects of bFGF-RBC/NP on cardiomyocyte apoptosis, we examined the apoptotic index by TUNEL staining in the heart tissues and quantitatively analyzed the fluorescent intensity of each group. The bFGF-RBC/NP groups exhibited lower fluorescent intensities in the treatment groups (Figures 6A,B). Additionally, we detected the expression of iNOS, which represented an endoplasmic reticulum stress-related marker that was related to cell apoptosis. Immunofluorescent staining of iNOS suggested a rapid increase in the CLP model, compared to the control group. Contrarily, bFGF-RBC/NP downregulated the iNOS expression (Figures 6C,D). Taken together, these results confirmed the excellent protective effects against the inflammatory response and apoptosis of bFGF-RBC/NP in sepsis-induced cardiac injury.

### *In Vivo* Antioxidant and Anti-Inflammatory Effects of bFGF-RBC/NP

**FIGURE 7:**
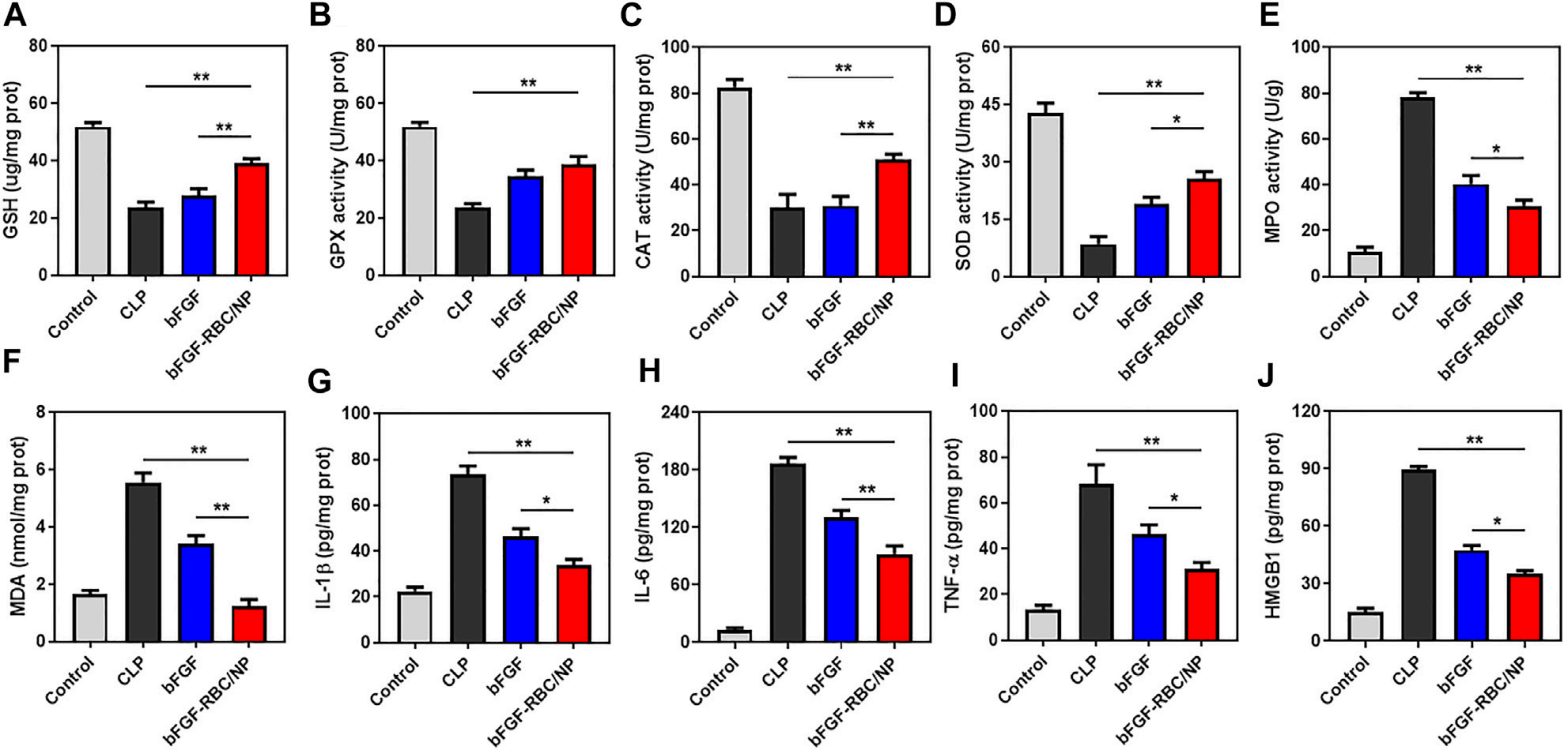
*In vivo* antioxidant and anti-inflammatory effects of bFGF-RBC/NP. The levels of **(A)** GSH, **(B)** GPX, **(C)** CAT, **(D)** SOD, **(E)** MPO, and **(F)** MDA in heart tissues. The contents of **(G)** IL-1β, **(H)** IL-6, **(I)** TNF-α, and **(J)** HMGB1 in heart tissues. Data are expressed as the mean ±SD (*n* = 3). *p < 0.05 and **p < 0.01.

Subsequently, we evaluated the antioxidant and anti-inflammatory effects of bFGF-RBC/NP in the sepsis-cardiac injury model. The cardiac tissues were obtained to measure related parameters. The results showed that bFGF-RBC/NP significantly enhanced the antioxidant GSH level and activities of fundamental antioxidant enzymes, including GPX, CAT, and SOD, in the heart during sepsis-cardiac injury. This indicated that the antioxidant capacity of the heart tissues improved, which was conducive to preventing further aggravation of cardiac injury (Figures 7A–D). In addition, bFGF-RBC/NP effectively reduced the MPO activity and MDA level that facilitated the recovery of cardiac injury (Figures 7E,F). Additionally, we detected IL-1β, IL-6, TNF-α, and HMGB1 protein levels in the heart tissue using ELISA kits. Distinctly, the secretion of these inflammatory cytokines was inhibited by bFGF-RBC/NP (Figures 7G–J). Collectively, our results demonstrated that bFGF-RBC/NP could attenuate sepsis-induced cardiac injury by antioxidative and anti-inflammatory effects and showed better therapeutic effects than free bFGF.

## Discussion

In this study, we designed and synthesized bFGF-RBC/NP, which could enhance the stability of bFGF. The RBC membrane further prolonged the circulation of bFGF-RBC/NP, thus increasing the potency of bFGF. Although emerging delivery strategies have been developed for bFGF drugs, we used biomimetic PLGA nanoparticles as a vehicle for bFGF in sepsis-induced cardiac injury.

Currently, cardiac dysfunction caused by sepsis, which is a leading cause of death in intensive care units, is associated with significant mortality with complicated pathogenesis (Angus et al., 2006). A landmark study has elucidated the mechanisms of myocardial dysfunction in sepsis, such as oxidative stress and inflammation, mainly caused by a dysregulated host response to infection (Singer et al., 2016). A range of strategies are attractive in theory; however, therapeutic approaches are limited for clinical use (Li et al., 2021a). Numerous studies have proven the benefits of bFGF in cardiovascular disease and sepsis, credited to their positive functions in angiogenesis and proliferation and their anti-inflammatory and antioxidative effects. As reported, biomimetic PLGA nanoparticles have been applied for the delivery of proteins, peptides, genes, vaccines, and antigens, owing to their biocompatibility, biodegradability, low immunogenicity, and good mechanical property (Jo et al., 2020). Considering the positive charge of bFGF, a strong electrostatic interaction would work between bFGF and PLGA, which is negatively charged (Shen et al., 2011). Bulk loading of bFGF into PLGA nanoparticles guaranteed the sustained and controlled release of bFGF, owing to the tight junctions among them. Sufficient evidence in the literature suggested that cell membrane proteins played a vital role in the immune-evasive function of RBCs, and CD47 is the most representative protein (Rodriguez et al., 2013). Successful cloaking of the RBC membrane endowed bFGF-NP with the ability to evade macrophage recognition and attributed to the numerous proteins on the RBC membrane, which might preliminarily explain the better therapeutic effects of bFGF-RBC/NP. bFGF-RBC/NP had a compact structure and smooth surface, with a size of approximately 200 nm. Additionally, it was negatively charged and had great biocompatibility and stability, which were suitable for intravenous injection (Wong et al., 2008).

Pathogenesis of sepsis-induced cardiac injury covered overwhelming aspects. Cardiomyocyte depression caused by circulating cytokines, such as TNF-α and IL-1β, was indicated by an *in vitro* test, which was responsible for myocardial dysfunction (Kumar et al., 1996). Persistent inflammatory activation might have a severe influence on cardiovascular conditions. Oxidative stress might contribute to myocardial dysfunction during sepsis (Haileselassie et al., 2017). Concentrating on this, we investigated the antioxidative and anti-inflammatory functions of bFGF-RBC/NP, both *in vitro* and *in vivo*. Our findings indicated that bFGF-RBC/NP showed synergistic antioxidative, apoptotic, and inflammatory benefits on cardiac treatment in sepsis, owing largely to the “core-shell” nanostructure and long circulation. More importantly, these beneficial effects by bFGF-RBC/NP were better than those of bFGF only.

## Conclusion

The successfully prepared biomimetic nanocarriers (bFGF-RBC/NP) had good properties, including suitable size, negative charge, sustained drug-release kinetics, great biocompatibility *in vitro*, and long blood circulation *in vivo*. Our study demonstrated that bFGF-RBC/NP pretreatment significantly alleviated oxidative damage, inhibited cell apoptosis, attenuated inflammatory response, and improved myocardial function. Therefore, bFGF-RBC/NP therapy can be considered a promising strategy for the treatment of cardiac injury in sepsis.

## Data Availability

The original contributions presented in the study are included in the article/Supplementary Material, further inquiries can be directed to the corresponding authors.
