# Supplementary material for: Red Blood Cell Membrane-Camouflaged PLGA Nanoparticles Loaded With Basic Fibroblast Growth Factor for Attenuating Sepsis-Induced Cardiac Injury
Source: Front Pharmacol. 2022 May 17;13:881320. doi: 10.3389/fphar.2022.881320 (PMC9152292; doi:10.3389/fphar.2022.881320)
Supplement: Supplementary file 1 [file DataSheet1.docx]

Supplementary materials

Red Blood Cell Membrane-Camouflaged PLGA Nanoparticles Loaded with Basic Fibroblast Growth Factor for Attenuating Sepsis-Induced Cardiac Injury

Xinze Li^1,4,*^, Guangliang Hong^1,4^, Guangju Zhao^1,4^, Hui Pei^1,4^, Jie Qu^1,4^, Changju Chun^3^, Zhiwei Huang^2,3,*^and Zhongqiu Lu^1,4,*^

^1^Department of Emergency, the First Affiliated Hospital of Wenzhou Medical University, Wenzhou 325035, China

^2^School of Pharmaceutical Sciences, Wenzhou Medical University, Wenzhou 325035, China.

^3^Research Institute of Pharmaceutical Sciences, College of Pharmacy, Chonnam National University, Gwangju 61186, Republic of Korea

^4^Wenzhou Key Laboratory of emergency and disaster medicine, Wenzhou 325035, China.

*** Correspondence:** lxzpharm@163.com (Xinze Li); hzwpharm@163.com (Zhiwei Huang); lzq_640815@163.com (Zhongqiu Lu)


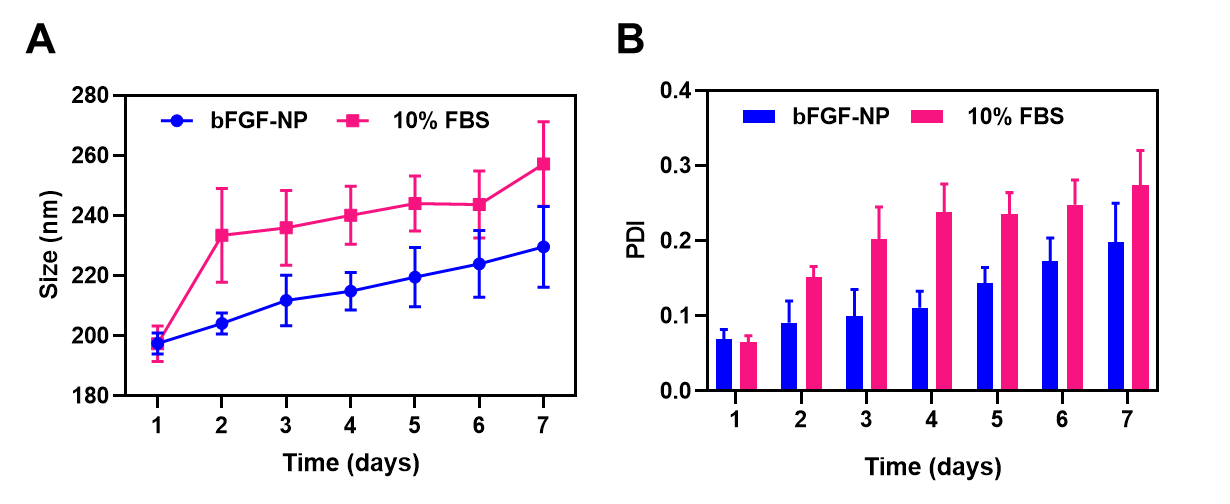


**Figure S1.** The (A) size and (B) PDI of bFGF-NP in PBS and PBS containing 10% FBS for 7 days. Data are expressed as the mean ± SD (n = 3).

**Figure S2.** The concentration of bFGF in blood from CLP mice after intravenous administration of bFGF (100 ug/kg), bFGF-NP (100 ug/kg) or bFGF-RBC/NP (100 ug/kg). Data are expressed as the mean ± SD (n = 3).
